# Mating success and body condition not related to foraging specializations in male fur seals

**DOI:** 10.1098/rsos.160143

**Published:** 2016-07-06

**Authors:** L. Kernaléguen, Y. Cherel, C. Guinet, J. P. Y. Arnould

**Affiliations:** 1 Geelong, School of Life and Environmental Sciences, Deakin University, Burwood, Victoria, Australia; 2 Centre d'Etudes Biologiques de Chizé, UMR 7372 du CNRS-Université de La Rochelle, BP 14, 79360 Villiers-en-Bois, France

**Keywords:** *Arctocephalus*, diet, fitness payoff, reproductive success, stable isotopes, territorial males

## Abstract

Individual specialization is widespread among wild populations. While its fitness consequences are central in predicting the ecological and evolutionary trajectories of populations, they remain poorly understood. Long-term individual foraging specializations occur in male Antarctic (*Arctocephalus gazella*) and Australian (*A. pusillus doriferus*) fur seals. Strong selective pressure is expected in these highly dimorphic and polygynous species, raising the question of the fitness payoffs associated with different foraging strategies. We investigated the relationship between individual isotopic niche (a proxy of foraging specialization), body size and condition, and an index of reproductive success (harem size) in territorial males. Individuals varied greatly in their skin and fur isotopic values reflecting a range of foraging strategies within the two populations. However, in both species, isotopic niche was not correlated to body size, condition or mating success (*R*^2^/*ρ* < 0.06). Furthermore, no foraging niche was predominant in either species, which would have indicated a substantial long-term fitness benefit of a particular strategy via a higher survival rate. These results suggest that the fitness consequences of a foraging strategy depend not only on the quality of prey and feeding habitat but also on an individual's hunting efficiency and skills.

## 1. Background

Within a population, individuals vary in many traits including their morphology, physiology, breeding status or learned abilities such that their optimal foraging strategy may differ, potentially leading to individual specializations [1]. While individual variation in resource use has been widely documented [1], less is understood about the consequences of such specialization. In environments where food is limited, foraging efficiency determines the quantity and quality of energy that can be allocated to growth, reproduction and survival. Furthermore, when targeting different resources, individuals might be exposed to different levels of threat such as predation [2] or pathogen exposure [3]. Hence, different feeding strategies could result in different fitness payoffs [4,5].

**Figure 1.**
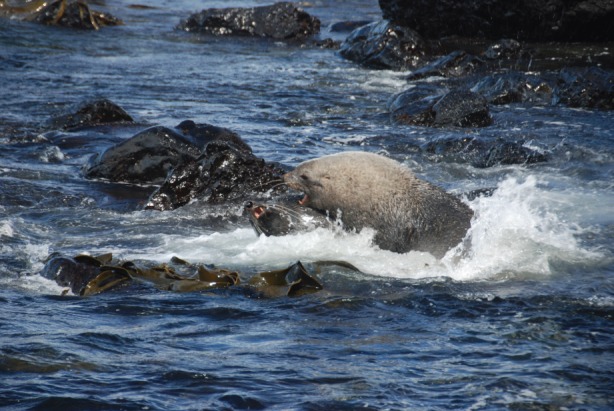
Territorial fight between two Antarctic fur seal bulls to gain access to females during the mating season, Kerguelen Islands. Photo credit: Laëtitia Kernaléguen.

In sexually dimorphic polygynous mating systems, only the largest, most dominant males have access to females such that breeding success can vary dramatically between individuals (figure 1). For example, as few as 3% of male northern elephant seals (*Mirounga angustirostris*) can be responsible for up to 92% of the mating observed within a breeding season [6]. As large body size provides advantages in male–male conflicts for the defence of territories or females, there is strong selection for increasing male size. Indeed, pinnipeds display the greatest size dimorphism in vertebrates, with males weighing up to 10 times the mass of females in some species [7]. Recent studies have demonstrated long-term individual dietary specialization in male Antarctic (*Arctocephalus gazella*) and Australian (*A. pusillus doriferus*) fur seals [8–10]. As territorial bulls are expected to be subject to strong selective pressure toward efficient foraging behaviour, they provide a unique opportunity to test the fitness consequences of individual specialization. Therefore, the aim of this study was to test the relationship between males' foraging strategy and reproductive success and body condition in these two fur seal species.

## 2. Material and methods

The study was conducted at the Antarctic fur seal Pointe Suzanne colony (49°26′S, 70°26′E), Kerguelen archipelago, during the 2013 mating season. Pointe Suzanne is a low-density colony spread along approximately 1 km of coastline, which consists of a narrow beach bordered by a small cliff surrounded by a plateau. Approximately 25% (*n* = 12), 100% (*n* = 38) and 100% (*n* = 24) of males seen within the same location for several consecutive days in the beach, plateau and hinterland, respectively, where individually paint-marked, using a brush fixed on a 2.5 m long pole. A relative index of mating success for each male was estimated from the total number of females present within the male's harem during its tenure duration. Number of females present in each harem was counted daily, from 5 December when the first pup was born, until the end of the mating season (31 December). However, harem sizes could not be monitored from 14 to 21 December due to logistic reasons.

Skin and fur biopsy samples were obtained from 69 and 73 males, respectively, using an 8 mm biopsy head. Biopsies were performed manually, with the biopsy head attached to a 2 m long pole. Length and body condition (surface/length) indices at arrival were assessed for 53 males using laser-metrics. Two parallel laser-pointers (30 mW Aussie Made Rifle Pistol Green Lasers Pointers, Telescopes and Astronomy, Ohalloran Hill, WA, Australia) were mounted 200 mm apart on a digital camera. Laser parallelism was checked before each photograph session at a distance of 25 m. All photographs were taken at distances less than 10 m, while males were in the prone position and perfectly perpendicular to the laser beams. Straight length index, from nose to tail, and the surface of males were estimated on photographs using the two laser beams as a scale, in Adobe Photoshop CS6.

Fieldwork was conducted on the Australian fur seal on Kanowna Island (39°10′S, 146°18′E), during the 2012 mating season. The Kanowna Island colony is a heterogeneous area varying primarily in elevation (i.e. access to water) [11]. Seven zones of contrasting quality have previously been described, covering 53% of the colony. The boundary of harems is not distinguishable in this higher density colony such that harem size was calculated as the ratio between the number of females and territorial males within each zone [11]. Males were individually identified from natural marks (e.g. scars, fur coloration). An index of relative mating success was calculated from 39 males across six zones, corresponding to 49% of territorial males breeding within these zones. Census of bulls and females were performed every 3 days, from 6 November until 16 December. Unusually high movement of females was observed on 13 December due to uncharacteristic early morning hot temperatures. Hence, data for this specific day were excluded. A hair sample was collected from these territorial males using an 8 mm biopsy head attached to an arrow launched by a crossbow (Sanlida Chase Wind 90 lbs).

The stable isotope niche of a predator provides a proxy of its foraging ecology, with δ^13^C and δ^15^N values documenting individual foraging habitat and trophic level, respectively [12]. While skin documents the feeding habits over the last few weeks prior to males arriving at the colony, fur is a metabolically inert tissue and reflects the isotopic signature of the diet during the last moult, seven to eight months prior to the breeding season. Prior to analysis, lipids were removed from skin samples using a cyclohexane solvent and only guard hairs were analysed. Replicate measurements of internal laboratory standards indicated isotopic measurement errors less than 0.10‰ for both isotopic ratios. Groups were compared using one-way ANOVA or Kruskal–Wallis test, and the percentage of isotopic niche overlap was calculated as the overlap of the standard ellipse area corrected for unbalanced sample sizes (SEAc) of each group, using the Bayesian ellipse-based metrics SIBER (Stable Isotope Bayesian Ellipses in R, [13]), using the SIAR package in R [13]. Correlation was tested using Pearson or Spearman's rank correlation, according to the normality of data. Statistics were performed using R v. 3.0.3.

## 3. Results

**Figure 2.**
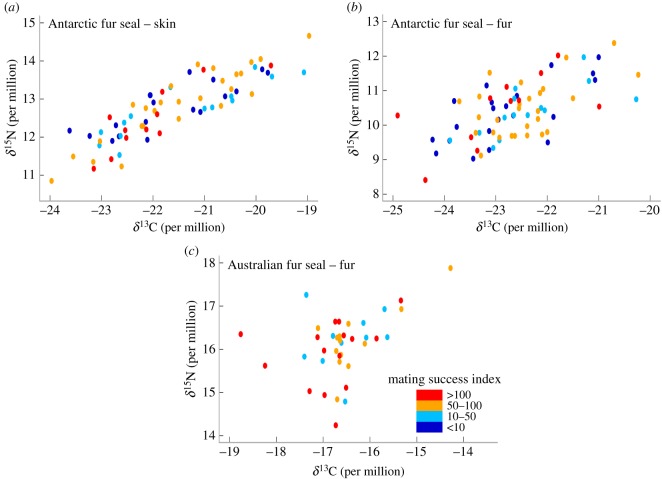
Skin and fur δ^13^C and δ^15^N values of territorial males in relation to their index of mating success.

Territorial male Antarctic fur seals varied greatly in size and body condition (table 1). Males tended to be smaller in the hinterland (*F*_2,50_ = 2.86, *p* = 0.066) but of similar length and condition in the beach and plateau (table 2). Tenure duration and the index of mating success were similar in the beach and plateau, and lower in the hinterland where harems are usually smaller and unstable (table 2). Males occupied a large isotopic niche (table 1), with no variation in skin or fur isotopic values between zones (all *F*_2,66_ < 2.66, *p* > 0.17, with pairwise SEAc overlap ranging between 89 and 92% and 68 and 88% for skin and fur samples, respectively). There were no relationships between length or body condition and male tenure duration or their index of mating success (all *ρ* < 0.03, *p* > 0.27, *n* = 53). Similarly, skin and fur δ^13^C and δ^15^N values were not correlated to length, body condition (all *R*^2^ < 0.06, *p* > 0.07, *n* = 53) or index of mating success (both *ρ* < 0.01, *p* > 0.33, *n* = 69/73; figure 2).

**Table 1.** Index of mating success, isotopic values and body size and condition of territorial male Antarctic and Australian fur seals. Results are mean ± s.d. (range).

	Antarctic fur seal	Australian fur seal
tenure duration (d)	27.9 ± 14.4 (4; 41)	34.0 ± 6.3 (12; 39)
overall number of females	52.7 ± 43.7 (0; 147)	94.3 ± 54.6 (44.6; 218.2)
fur δ^13^C (‰)	−22.6 ± 0.9 (−24.9; −20.2)	−16.5 ± 1.0 (−18.4; −12.2)
fur δ^15^N (‰)	10.4 ± 0.8 (8.4; 12.4)	16.1 ± 0.7 (14.2; 17.8)
skin δ^13^C (‰)	−21.6 ± 1.2 (−24.0; −19.0)	
skin δ^15^N (‰)	12.8 ± 0.8 (10.9; 14.7)	
length index (cm)	147 ± 9 (121; 167)	
body condition index (cm)	24 ± 2 (19; 28)

**Table 2.** Index of mating success, isotopic values and body size and condition of territorial male Antarctic fur seals breeding on the beach, plateau and hinterland.

	beach	plateau	hinterland
tenure duration (d)*		30.8 ± 13.8^a^	16.8 ± 10.3^b^
overall number of females*	64.8 ± 36.2^a^	67.1 ± 45.1^a^	23.8 ± 29.4^b^
fur δ^13^C (‰)	−22.5 ± 1.0	−22.5 ± 1.0	−22.8 ± 0.8
fur δ^15^N (‰)	10.2 ± 0.7	10.6 ± 0.9	10.2 ± 0.7
skin δ^13^C (‰)	−21.7 ± 1.3	−21.5 ± 1.2	−21.7 ± 1.2
skin δ^15^N (‰)	12.6 ± 1.0	12.8 ± 0.8	12.7 ± 0.8
length index (cm)	149 ± 5	149 ± 9	142 ± 11
body condition index (cm)	24 ± 2	24 ± 2	23 ± 2

Significant differences between zones are indicated (*p < 0.05), with superscripts representing homogeneous subsets.

Tenure duration results are not reported for the beach as males were not sampled uniformly during the mating season, artificially increasing the average tenure duration of males breeding in this zone.

Male Australian fur seals varied greatly in harem size, tenure duration and fur δ^13^C and δ^15^N values (table 1). The index of mating success was not correlated with individual isotopic niche (both *ρ* < 0.02, *p* > 0.41, *n* = 38/39; figure 2). Fur isotopic values were similar across all zones (both *H*_7_ < 8.9, *p* > 0.26 with pairwise SEAc overlap ranging between 47 and 100%; table 3) indicating body size and condition, assessed by the location of breeding territories [11], were not related to isotopic niche.

**Table 3.** Fur isotopic values of male Antarctic fur seals holding a territory in six of the seven zones described by Lourie *et al.* [11].

	*N*	fur δ^13^C (‰)	fur δ^15^N (‰)
zone 1	10	−16.9 ± 1.0	16.0 ± 0.7
zone 2	5	−16.6 ± 0.1	16.1 ± 0.6
zone 3	5	−16.4 ± 0.7	15.8 ± 1.0
zone 4	8	−16.4 ± 0.9	16.2 ± 0.9
zone 5	2	−14.4 ± 3.1	16.2
zone 6	9	−16.5 ± 0.7	16.2 ± 0.7

## 4. Discussion

While the skin and fur samples only measured two narrow time-periods in trophic niche, previous studies in these species have indicated that short-term inter-individual variation in blood isotopic values was correlated with long-term specialization revealed in whisker isotopic signatures [8–10,14]. As expected [8], territorial male Antarctic fur seals occupied a wide isotopic niche. While part of the isotopic variation may be attributed to inter-individual physiological variation, δ^13^C values were characteristic of males foraging along a latitudinal gradient, from Antarctic to subtropical waters. δ^15^N values were highly correlated to δ^13^C values, indicating that males altered their diet depending on their foraging habitat and the associated prey. Results indicate bulls fed most probably on the Antarctic krill (*Euphausia superba*) in Antarctic waters where this resource is highly abundant and switched to myctophid fish and oceanic squids when foraging in northern areas [8]. Despite Australian fur seals having a much reduced habitat range compared with Antarctic fur seals [10,15], territorial males of this species also exhibited a wide isotopic niche. Interestingly, the range of isotopic values was much larger than previously shown for adult females and, more importantly, small but sexually mature males [10,14,15], suggesting ontogenetic variation in foraging niche associated with reproduction in males.

Body size is expected to confer an advantage in male–male conflicts and fasting abilities in dimorphic, capital breeding males [6]. Accordingly, larger male Australian fur seals hold territories in higher quality habitats, which are characterized by earlier occupancies, greater female densities and harem sizes, and are occupied by larger breeding females [11]. Surprisingly, however, contrasting results were found for Antarctic fur seals. Males tended to be smaller in the hinterland where harems are smaller and unstable. However, tenure duration and the index of mating success were not correlated to body size or condition. This could be due to the Pointe Suzanne being a low-density colony and that competition for territories is low enough that individual differences in motivation and personality or that some level of female mate choice influence male mating success [16].

Males of such highly polygynous, sexually dimorphic species are expected to be subject to strong selective pressure toward efficient foraging behaviour. Hence, a clear and strong pattern would be expected if specializations differed in their respective fitness payoffs. However, individual foraging niche was not correlated to length, body condition or mating success, in either species. It is possible that the consequences of specialization occur at an earlier stage in the males' life (i.e. survival) and that territorial males across the colonies may already represent a reduced set of good quality individuals. However, territorial males of both species occupied a very large isotopic niche, indicating that a wide range of foraging strategies allows males to reach breeding age. Furthermore, no foraging niche was predominant in either species (figure 2), which would have indicated a substantial long-term fitness benefit of a particular dietary strategy via a higher survival rate [7]. Alternatively, the use of a fairly high number of habitats has the consequence of diminishing consumer density in a given habitat, reducing potential inter-individual competition for trophic resources.

Contrasting results have been found on the reproductive consequences of individual specialization in different taxa [4,5,7,17]. The main drivers maintaining individual variability appear to play an important role in predicting the occurrence of a relationship. Where dietary specialization is a heritable factor, natural selection should only maintain the most efficient strategies in the population, and all specializations should confer similar fitness payoffs [7]. By contrast, variation in fitness consequence would be expected where social dominance maintains suboptimal strategies in the population [5] or when individual specialization is driven by disruptive selection or fluctuating selection on specialists in time or space [18,19].

While the majority of studies investigate the impact of diet and habitat selection, fitness consequences might not vary depending on the type of resources used but on the individual's behaviour and hunting abilities. Indeed, the main driver of individual specialization is individual variability, notably in morphology, physiology, experience or skills [1]. As conspecifics differ in traits and characteristics, it is expected that the fitness payoff of a specific foraging strategy should vary between individuals, and that a range of foraging niches should confer fitness advantages depending on individual characteristics. Accordingly, in this study, males occupying the same isotopic niche varied greatly in their body size, condition and mating success. This suggests that the fitness consequence of a foraging strategy depends not only on the quality of prey and feeding habitat but also on an individual's hunting efficiency and skills (i.e. not only what an individual eats but also how much).

## Data accessibility

Data available from the Dryad Digital Repository, http://dx.doi.org/10.5061/dryad.792gj.

## References

[1] Bolnick DI, Svanback R, Fordyce JA, Yang LH, Davis JM, Hulsey CD, Forister ML 2003 The ecology of individuals: incidence and implications of individual specialization. Am. Nat. 161, 1–28. (doi:10.1086/343878) 12650459 10.1086/343878

[2] Darimont CT, Paquet PC, Reimchen TE 2007 Stable isotopic niche predicts fitness of prey in a wolf-deer system. Biol. J. Linn. Soc. 90, 125–137. (doi:10.1111/j.1095-8312.2007.00716.x)

[3] Johnson CK, Tinker MT, Estes JA, Conrad PA, Staedler M, Miller MA, Jessup DA, Mazet JA 2009 Prey choice and habitat use drive sea otter pathogen exposure in a resource-limited coastal system. Proc. Natl Acad. Sci. USA 106, 2242–2247. (doi:10.1073/pnas.0806449106) 19164513 10.1073/pnas.0806449106 PMC2650139

[4] Vander Zanden HB et al. 2014 Foraging areas differentially affect reproductive output and interpretation of trends in abundance of loggerhead turtles. Mar. Biol. 161, 585–598. (doi:10.1007/s00227-013-2361-y)

[5] Marra PP, Hobson KA, Holmes RT 1998 Linking winter and summer events in a migratory bird by using stable-carbon isotopes. Science 282, 1884–1886. (doi:10.1126/science.282.5395.1884) 9836637 10.1126/science.282.5395.1884

[6] Le Boeuf B, Reiter J 1988 Lifetime reproductive success in northern elephant seals. In Reproductive success: studies of individual variation in contrasting breeding systems. (ed. Clutton-Brock TH). Chicago, IL: University of Chicago Press.

[7] Authier M, Bentaleb I, Ponchon A, Martin C, Guinet C 2012 Foraging fidelity as a recipe for a long life: foraging strategy and longevity in male southern elephant seals. PLoS ONE 7, e32026 (doi:10.1371/journal.pone.0032026) 22505993 10.1371/journal.pone.0032026 PMC3323586

[8] Cherel Y, Kernaléguen L, Richard P, Guinet C 2009 Whisker isotopic signature depicts migration patterns and multi-year intra- and inter-individual foraging strategies in fur seals. Biol. Lett. 5, 830–832. (doi:10.1098/rsbl.2009.0552) 19793740 10.1098/rsbl.2009.0552 PMC2828010

[9] Kernaléguen L, Cazelles B, Arnould JPY, Richard P, Guinet C, Cherel Y 2012 Long-term species, sexual and individual variations in foraging strategies of fur seals revealed by stable isotopes in whiskers. PLoS ONE 7, e32916 (doi:10.1371/journal.pone.0032916) 22431988 10.1371/journal.pone.0032916 PMC3303799

[10] Kernaléguen L, Cherel Y, Knox TC, Baylis AM, Arnould JP 2015 Sexual niche segregation and gender-specific individual specialization in a highly dimorphic marine mammal. PLoS ONE 10, e0133018 (doi:10.1371/journal.pone.0133018) 26244371 10.1371/journal.pone.0133018 PMC4526469

[11] Lourie HJ, Hoskins AJ, Arnould JP 2014 Big boys get big girls: factors influencing pupping site and territory location in Australian fur seals. Mar. Mamm. Sci. 30, 544–561. (doi:10.1111/mms.12056)

[12] Kelly JF 2000 Stable isotopes of carbon and nitrogen in the study of avian and mammalian trophic ecology. Can. J. Zool. 78, 1–27. (doi:10.1139/z99-165)

[13] Jackson AL, Inger R, Parnell AC, Bearhop S 2011 Comparing isotopic niche widths among and within communities: SIBER–Stable Isotope Bayesian Ellipses in R. J. Anim. Ecol. 80, 595–602. (doi:10.1111/j.1365-2656.2011.01806.x) 21401589 10.1111/j.1365-2656.2011.01806.x

[14] Kernaléguen L et al. 2016 From video recordings to whisker stable isotopes: a critical evaluation of timescale in assessing individual foraging specialization in Australian fur seals. Oecologia 180, 657–670. (doi:10.1007/s00442-015-3407-2) 26233674 10.1007/s00442-015-3407-2

[15] Arnould JPY, Cherel Y, Gibbens J, White JG, Littnan CL 2011 Stable isotopes reveal inter-annual and inter-individual variation in the diet of female Australian fur seals. Mar. Ecol. Prog. Ser. 422, 291–302. (doi:10.3354/meps08933)

[16] Boness DJ 1991 Determinants of mating systems in the Otariidae (Pinnipedia). In The behaviour of pinnipeds. (ed. Renouf D), pp. 1–44. Berlin, Germany: Springer.

[17] Woo KJ, Elliott KH, Davidson M, Gaston AJ, Davoren GK 2008 Individual specialization in diet by a generalist marine predator reflects specialization in foraging behaviour. J. Anim. Ecol. 77, 1082–1091. (doi:10.1111/j.1365-2656.2008.01429.x) 18624834 10.1111/j.1365-2656.2008.01429.x

[18] Van De Pol M, Brouwer L, Ens BJ, Oosterbeek K, Tinbergen JM 2010 Fluctuating selection and the maintenance of individual and sex-specific diet specialization in free-living oystercatchers. Evolution. 64, 836–851. (doi:10.1111/j.1558-5646.2009.00859.x) 19804401 10.1111/j.1558-5646.2009.00859.x

[19] Cucherousset J, Acou A, Blanchet S, Britton JR, Beaumont WR, Gozlan RE 2011 Fitness consequences of individual specialization in resource use and trophic morphology in European eels. Oecologia. 167, 75–84. (doi:10.1007/s00442-011-1974-4) 21455773 10.1007/s00442-011-1974-4

